# Transcriptomic diversity in seedling roots of European flint maize in response to cold

**DOI:** 10.1186/s12864-020-6682-1

**Published:** 2020-04-15

**Authors:** Felix P. Frey, Marion Pitz, Chris-Carolin Schön, Frank Hochholdinger

**Affiliations:** 1 Institute of Crop Science and Resource Conservation, Crop Functional Genomics, University of Bonn, Bonn, Germany; 2 Department of Plant Breeding, Technische Universität München, Freising, Germany

**Keywords:** Abiotic stress, Cold, Doubled haploids, Flint, Landrace, Maize, RNA-seq, Root, Transcriptome

## Abstract

**Background:**

Low temperatures decrease the capacity for biomass production and lead to growth retardation up to irreversible cellular damage in modern maize cultivars. European flint landraces are an untapped genetic resource for genes and alleles conferring cold tolerance which they acquired during their adaptation to the agroecological conditions in Europe.

**Results:**

Based on a phenotyping experiment of 276 doubled haploid lines derived from the European flint landrace “Petkuser Ferdinand Rot” diverging for cold tolerance, we selected 21 of these lines for an RNA-seq experiment. The different genotypes showed highly variable transcriptomic responses to cold. We identified 148, 3254 and 563 genes differentially expressed with respect to cold treatment, cold tolerance and growth rate at cold, respectively. Gene ontology (GO) term enrichment demonstrated that the detoxification of reactive oxygen species is associated with cold tolerance, whereas amino acids might play a crucial role as antioxidant precursors and signaling molecules.

**Conclusion:**

Doubled haploids representing a European maize flint landrace display genotype-specific transcriptome patterns associated with cold response, cold tolerance and seedling growth rate at cold. Identification of cold regulated genes in European flint germplasm, could be a starting point for introgressing such alleles in modern breeding material for maize improvement.

## Background

Maize displays the most widespread geographical distribution of all major crop species [1] with an annual grain harvest of 1135 million tons [2]. In the EU-28 countries, maize is grown second only to wheat by production [3]. Although maize has been adapted to a variety of environmental conditions, traits such as disease, insect resistance and abiotic stress tolerance can be further improved in elite germplasm subjected to a rapidly changing climate [4]. Since the introduction of maize in Europe, geographical separation and natural as well as human selection led to a diversification of landraces. Molecular analyses discovered that traditional flint corn (*Zea mays var. indurata*) populations of Northern Europe have major contributions from North American flints, which were introduced to Europe during the sixteenth century [5, 6]. Adaptations to the North and Central European climate included the development of a shorter growing cycle to avoid cold temperatures during the growth period and as another strategy, higher tolerance to cold temperatures [7]. A high genetic diversity, including favorable alleles to improve elite germplasm are present in European flint populations [4]. In the common dent x flint hybrids, the flint line represents in most instances the cold tolerant parent. However, to date, variation for cold tolerance in elite hybrids is scarce and maize is highly cold sensitive [8].

Due to its tropical origin, the optimum temperature for maize growth ranges from 21 to 27 °C [9]. Suboptimal temperatures decrease the capacity for biomass production and lead to growth retardation. Upon exposure to temperatures below 10 °C, which often occur at sowing time in Central and Northern Europe, cellular and tissue injuries may cause irreversible damage and may result in plant death [9]. Response to cold stress in maize has been studied broadly and many affected pathways have been identified, revealing the complexity of cold stress response. The immediate reactions to cold involve the decrease of CO_2_ assimilation and the down-regulation of photosynthetic electron transport in leaves, inhibiting photosynthesis [9–11]. Cell cycle duration and cell proliferation are reduced [12] and cell-wall organization is changed [13]. Furthermore, chilling activates different defense mechanisms. Antioxidant production and activity are altered as a result of increasing levels of reactive oxygen species (ROS) [14]. Changes in gene expression under cold stress include the repression of photosynthesis related genes. An induction is observed in genes related to transcription, phosphorylation, cell-wall organization and expression regulation [13]. Induced regulators are for example many phytohormones [15] and among those in particular salicylic acid (SA) and abscisic acid (ABA) [16, 17]. The impact of cold temperatures on root development and function has been less explored. One effect of cold temperatures is the reduction of hydraulic conductance of roots [18], which leads to water deficit of the plants under cold stress. Maize seedlings can acclimatize within 24 h which results in recovery of hydraulic conductance [19]. Further, the lengths of elongation zones and root growth-rates are reduced under cold stress thus affecting root architecture. In particular, the branching angles between primary and lateral roots are reduced upon cold stress [20].

To ensure high yield in temperate climates, a good early seedling vigor during cold temperatures is important. In Germany, maize is typically sown between mid-April and May, where temperatures regularly drop below 10 °C [21]. However, early sowing is advised by agricultural consultants in colder regions [22]. This strategy improves the performance throughout the year because the maize plants benefit from a longer vegetation period, improved vegetative growth and earlier ripening and harvest times. By early sowing, plants can avoid summer drought during flowering and ripening [23, 24]. Current agronomic strategies to reduce chilling effects in maize involve adaptation of sowing time and soil management such as preparation of a fast warming seedbed or mulching [14]. Breeding for cold tolerance during early development will also be important for no-tilling conservation agriculture, where soil warming is slower [25]. Therefore, inclusion of maize varieties with cold tolerance during early development will be important for environmentally protective agricultural practices.

Maize landraces are a rich source of favorable alleles for broadening the genetic basis of elite germplasm [4]. We hypothesized that European maize landraces display substantial variation for cold tolerance thus carrying beneficial alleles for this trait which might not be present in elite material. In this study, the transcriptomic response of pre-selected doubled haploid (DH) lines derived from the European flint landrace “Petkuser Ferdinand Rot” towards cold treatment was evaluated towards the goal to improve cold tolerance. DH technology is an efficient method to generate homozygous DH inbred lines by chromosome doubling of haploid cells [26].

The aims of this study were to identify genes associated with (i) the general response to cold treatment (ii) cold tolerance and (iii) seedling growth at cold conditions.

## Results

### Identification of cold tolerant and susceptible maize genotypes from doubled haploid lines derived from the flint landrace Petkuser Ferdinand rot

European elite maize germplasm shows only limited variation for the response to cold stress during early seedling development whereas European flint landraces harbor a high genetic diversity for cold tolerance. To untap this underutilized genetic resource, we screened 276 doubled haploid (DH) lines induced from the flint landrace ‘Petkuser Ferdinand rot’ for the growth response of primary roots to cold.

The primary root of maize was assessed in this study because it is the first organ which emerges after germination. Low soil temperatures at early stages of root development can negatively affect early seedling vigor which is important to ensure high yield in temperate climates. Moreover, the simple structure of the seedling primary root allowed to define the duration of cold stress treatment for the RNA-seq experiments during which no morphological changes of the root were monitored.

**Fig. 1:**
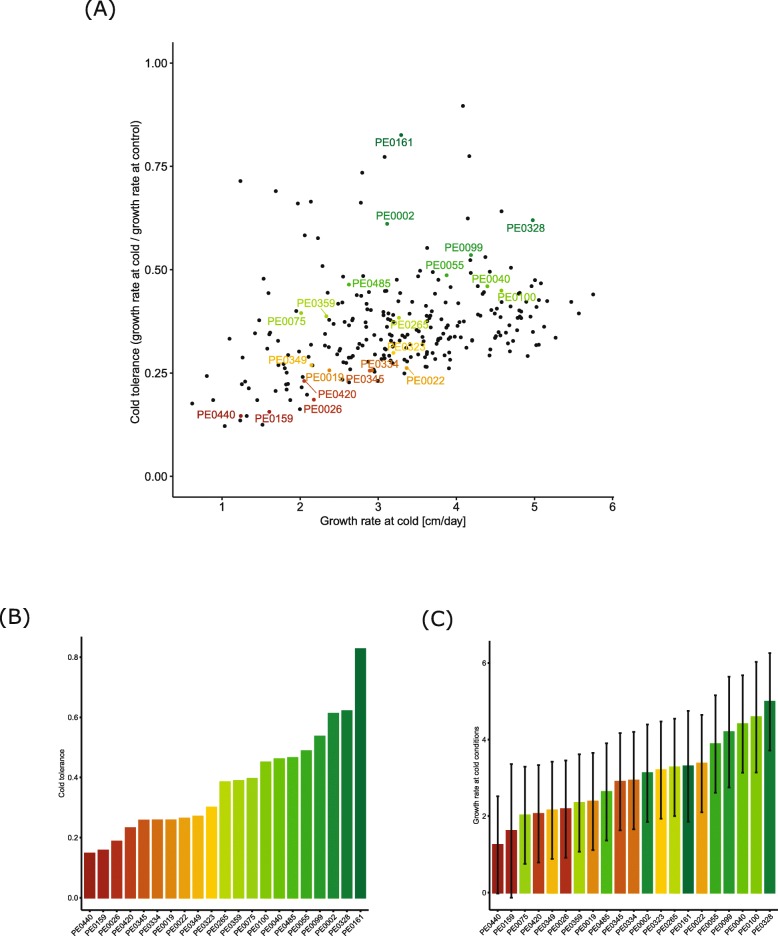
Cold tolerance and growth rate of maize primary roots at cold conditions. **a** Cold tolerance as ratio of root growth rate at control versus cold conditions (y-axis) and root growth rate at cold conditions (x-axis) of 276 DH-lines. Selected genotypes are colored according to ranking by cold tolerance values from tolerant (dark green) to susceptible (red). **b** Bar chart displaying the cold tolerance of the 21 tolerant and susceptible lines subjected to downstream RNA-seq experiments. **c** Genotypes from Fig. 1b sorted according to their growth at cold measured in cm/day, error bars represent standard errors of measurements

In this study we defined cold tolerance as the ratio of growth rate of the primary root at cold versus growth rate of the primary root at control conditions. As a second measure we defined growth rate at cold as absolute growth values under cold conditions in cm/day. These two measures are plotted in Fig. 1a. Cold tolerance and growth rate at cold were correlated significantly (*p* < 0.001). However, the coefficient of the correlation between the two traits in the DH-population was low with *r*  = 0.22.

Based on the results of this phenotyping experiment, we selected ten cold susceptible (yellow to orange features) and eleven cold tolerant DH-lines (green features) of the 276 genotypes for further analyses based on seed availability (Fig. 1a). The ranking of the genotypes with respect to cold tolerance (Fig. 1b) differed from the ranking of genotypes with respect to the root growth rate under cold conditions (Fig. 1c).

### Transcriptome profiling of cold tolerant and susceptible maize DH lines

Subsequently, we surveyed how the diversity for cold tolerance is reflected in the transcriptomic landscape of the primary roots in the selected DH lines. The transcriptomes of the eleven cold tolerant and ten cold susceptible lines were investigated after control and cold treatment in four biological replicates per genotype by treatment combination.

The RNA-Seq experiments yielded on average ~ 36 million 100 bp paired-end reads per sample (Table S1). The sequencing data has been deposited in the NCBI sequencing read archive (SRA; http://www.ncbi.nlm.nih.gov/sra; BioProject accession number PRJNA556806). Among those, on average 75% of the trimmed high-quality reads mapped at unique positions in the gene set of the maize B73 reference genome with 46,272 predicted coding and non-coding gene models (AGPv4 release 36) (Table S1).

We considered a gene active in a genotype if we detected on average ≥ 1 fragment per million reads (FPM) across all eight biological replicate samples of a genotype. The number of active genes ranged from 19,917 in PE0040 to 21,011 in PE0075 (Figure S1). Overall, 24,448 different genes were active in at least one genotype while 17,204 genes represented the core transcriptome, i.e. genes active in all 21 genotypes.

### Kinship relationship among the surveyed panel of maize DH lines

**Fig. 2:**
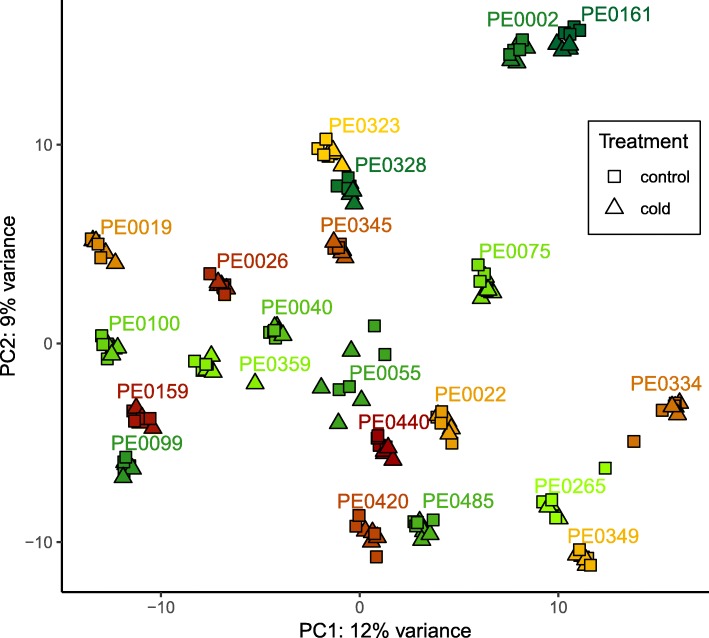
Principal component analysis assessing the transcriptomic relationships among the 21 tested maize DH lines under cold and control conditions. All RNA-seq samples were plotted in two dimensions representing the first and second principal component of the data. Cold tolerant genotypes are depicted with green colors, susceptible genotypes in red and orange tones. Color code according to Fig. 1

We determined the transcriptomic relationships among the 21 tested maize DH lines under cold and control conditions by a principal component analysis (PCA). In the PCA, the two principal components PC1 and PC2 explained 21% of the total variance (Fig. 2). The samples subjected to control and cold treatment clustered closely together, respectively for each genotype, indicating small overall transcriptomic differences between cold and control treatment. The very cold tolerant genotypes PE0161 and PE0002 clustered closely together and were clearly separated from the other genotypes (Fig. 2). We did not observe separation of the remaining tolerant versus susceptible genotypes. The transcriptomic relationship of the surveyed samples was in all instances mainly determined by the genotype and to a smaller extend by the treatment.

### Three types of transcriptomic responses to cold

**Fig. 3:**
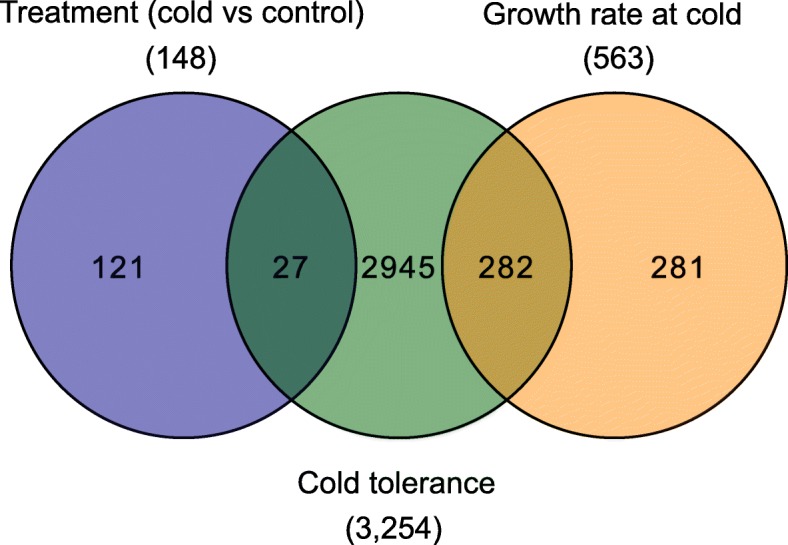
Venn diagram of the number of differentially expressed genes with FDR < 5% and |log_2_FC| > 1 for the factors treatment (cold vs. control), cold tolerance, and growth rate at cold. Numbers show overlaps of genes between different classes and uniquely differentially expressed genes for each class

We identified three types of transcriptomic responses associated with cold stress including treatment, cold tolerance and growth rate at cold (Fig. 3). Treatment, cold tolerance and the interaction between these two were determined with model 1 (see Methods). We identified genes which were differently expressed for treatment and cold tolerance, while for the interaction term no genes were found to be differentially expressed.

To investigate the gene expression changes of the studied genotypes associated with their different growth rates at cold conditions we applied model 3 (see Methods), where we included only samples from the cold treatment.

Finally, model 2 was applied to break down the transcriptomic response associated with the treatment effect (model 1) on the genotype level. To this end, we applied model 2 to each genotype in a separate analysis with the factor treatment alone. Thus, we were able to determine dependence of gene expression on the treatment effect for each genotype and refine the results of model 1.

Differentially regulated genes were computed by pairwise contrasts in the case of two factor levels or significances of continuous factors in the cases of quantitative traits, i.e. cold tolerance and growth rate at cold (FDR < 5% and |log_2_FC| > 1).

First, 148 genes differentially expressed upon cold treatment irrespective of the genotype (factor *t*_j_, model 1, see Methods). Second, 3254 genes, which were differently expressed with increasing cold tolerance irrespective of the expression differences between treatments (factor *c*_*i*_, model 1, see Methods) and third 563 genes associated with genotypic growth at cold conditions (factor *g*_*i*_ model 3, see Methods). No gene was identified as differentially expressed with respect to the interaction effect (*(ct)*_*ij*_ model 1, see Methods). In total, 27 genes were shared between the treatment and cold tolerance effect and 282 genes where differentially expressed with respect to cold tolerance and growth rate at cold. No gene was shared between treatment and growth rate at cold.

**Fig. 4:**
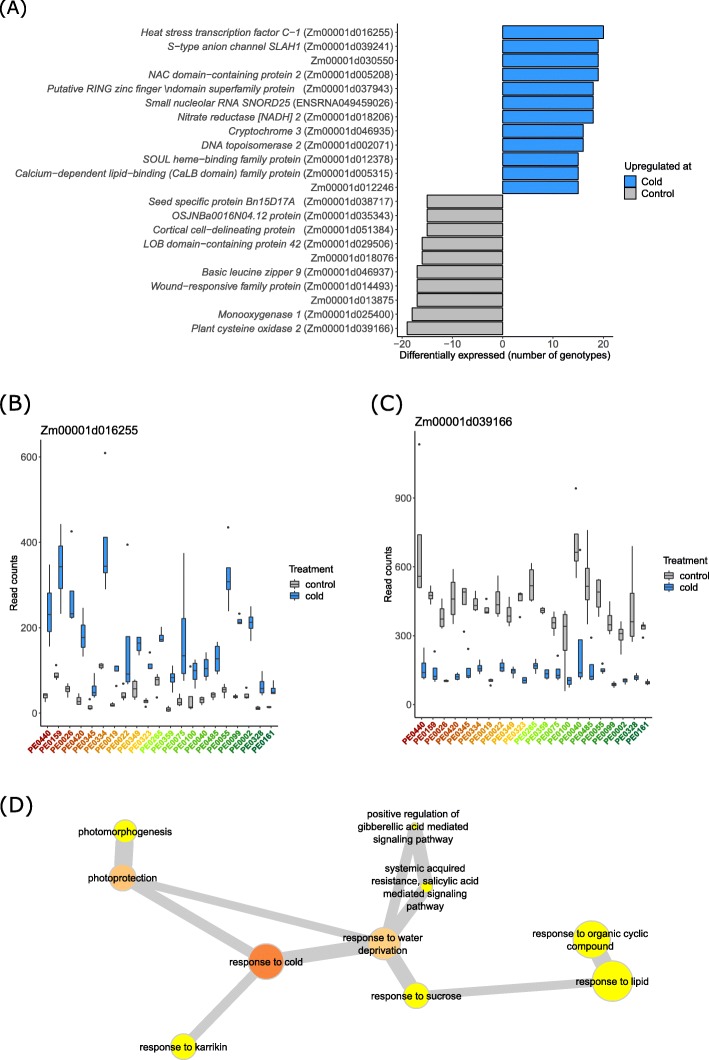
Genes differentially expressed upon cold treatment. **a** Genes preferentially expressed in either cold or control treated plants in ≥15 genotypes (FDR < 5% and |log_2_FC| > 1). **b** The *heat stress transcription factor C-1* (Zm00001d016255) was significantly upregulated upon cold treatment in 20 of 21 genotypes. **c** The *cysteine oxidase 1* (Zm00001d039166) was downregulated at cold treatment in 19 of 21 genotypes. **d** Network graph of enriched (*p* < 0.01) GO terms in the set of differentially expressed genes by treatment effect. Node size represents frequency of the GO term in the underlying database (the smaller the more specific). Node fill color represents the log_10_ of the enrichment *p*-value (the darker, the more enriched). Highly similar GO terms are linked by edges (grey lines), where edge width indicates the degree of similarity

Among the 148 genes associated with the treatment effect (Table S2), ten genes were downregulated in ≥15 genotypes and 12 genes were upregulated in ≥15 genotypes (model 2, see Methods, Fig. 4a, Table S3). The genes with the highest number of genotypes with differential expression upon cold treatment were a *heat stress transcription factor C-1* (Zm00001d016255) which was upregulated in 20 genotypes (Fig. 4b) and a *plant cysteine oxidase 2* (Zm00001d039166), which was downregulated in 19 genotypes (Fig. 4c).

Gene ontology (GO) term enrichment analysis with the 148 treatment-effect genes yielded 59 significantly (*p* < 0.05) enriched GO terms (Table S4). We identified a connected network of significantly (*p* < 0.01) enriched GO terms in the set of treatment-associated genes which was related to the response to cold, water deprivation and different organic compounds as well as with light-dependent processes and hormone signaling (Fig. 4d).

**Fig. 5:**
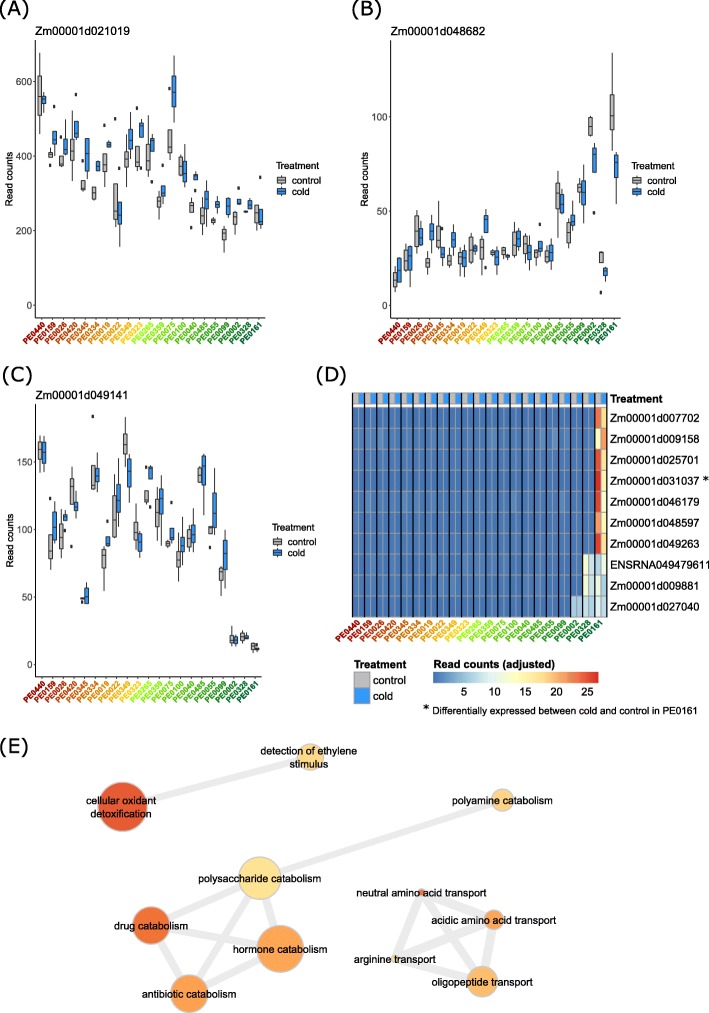
Examples of gene expression patterns associated with cold tolerance. **a** The *bHLH-transcription factor 136* gene (Zm00001d021019) showed gradually decreasing expression with increasing cold tolerance of genotypes, **b** the *GRAS-transcription factor 82* gene (Zm00001d048682) showed gradually increasing expression in line with increasing cold tolerance of genotypes. **c** The *Aux/IAA-transcription factor 14* (Zm00001d049141) was downregulated in the three most cold tolerant genotypes. Read counts in (A), (B) and (C) were normalized with plotCounts(). **d** Heat map of genes uniquely expressed in most tolerant one, two or three DH-lines (see Table S6). Values of each sample represent mean across four replicates/total mean across all samples. A gene with unknown function (Zm00001d031037) was significantly differently expressed between cold and control in DH-line PE0161. Read counts were adjusted by dividing the sample mean by the total mean across all samples. **e** Network graph of enriched (*p* < 0.01) GO terms in the set of differentially expressed genes by cold tolerance. For node size, fill color and edges refer to scale and description in Fig. 4d

The 3254 genes associated with cold tolerance (Table S5) displayed a gradient of gene expression along the susceptible versus tolerant genotypes Fig. 5a-c. Some genes displayed a consistent trend of gene expression change along the susceptible to tolerant genotypes (Fig. 5a-b). For instance, a gene encoding a *bHLH-transcription factor 136* (Zm00001d021019) (Fig. 5a) displayed a decrease in expression with increasing cold tolerance whereas the increase of expression of a *GRAS-transcription factor 82* gene (Zm00001d048682) correlated in general with increasing cold tolerance of genotypes (Fig. 5b). Other genes of that set displayed more pronounced differences between a subset of the susceptible and tolerant genotypes. For instance, the *Aux/IAA-transcription factor 14* gene (Zm00001d049141) displayed relatively low expression in the three most tolerant genotypes (Fig. 5c).

As a subset of the 3254 genes differentially expressed with increasing cold tolerance, we identified ten genes, which were exclusively expressed (FPM ≥1) in the single most tolerant (PE0161, seven genes) or the two most tolerant (PE0161, PE0328, two genes) or the three most tolerant (PE0161, PE0328, PE0002, one gene) genotypes (Fig. 5d, Table S6). Among the seven genes uniquely expressed in genotype PE0161 the gene of unknown function, Zm00001d031037 displayed a particularly high expression of > 1000 reads at control conditions and was significantly downregulated under cold conditions (*p* < 0.05; log_2_FC = − 0.82; average reads = 44; Fig. 5d). This was the only of the ten genes exclusively expressed in the most tolerant genotypes which showed differential expression between cold and control treatment in a genotype.

In the set of 3254 differentially expressed genes for the cold tolerance effect, we identified 64 enriched GO terms (Table S7). We detected connected networks of GO terms associated with amino acid transport, catabolic processes and cellular oxidant detoxification (Fig. 5e).

**Fig. 6:**
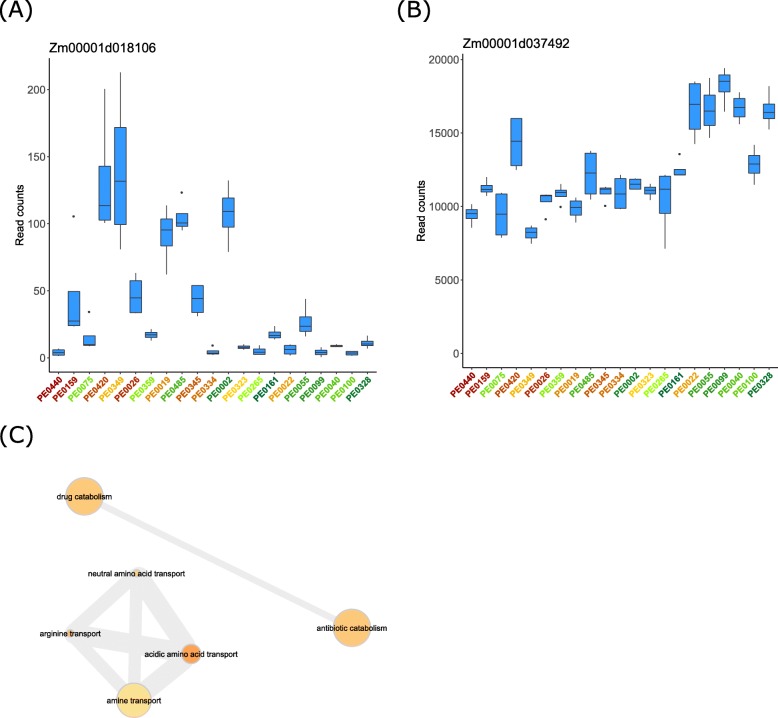
**a** and **b** Examples of gene expression patterns associated with root growth under cold conditions read counts were normalized with plotCounts(). Genotypes are sorted according to their growth under cold conditions (see Fig. 1c). **a** The *EXORDIUM* gene (Zm00001d018106) displayed in general lower expression levels in genotypes with faster root growth at cold conditions. **b** The *pyrophosphate-energized vacuolar membrane proton pump 1* gene (Zm00001d037492) displayed higher expression levels in genotypes with faster root growth at cold conditions. **c** Network graph of enriched (*p* < 0.01) GO terms in the set of differentially expressed genes expression patterns associated with root growth under cold conditions. For node size, fill color and edges refer to scale and description in Fig. 4d

The ranking of the genotypes with respect to growth rate at cold conditions was different from the order with respect to cold tolerance, where the y-axis shows growth rate at cold values, cold tolerance is represented by colors with green as cold tolerance and red as cold susceptible (Fig. 1b and c). We detected 563 genes with expression patterns associated with root growth under cold conditions (Table S8). Among those, a gene encoding a *EXORDIUM protein* (Zm00001d018106) displayed in general low expression in faster growing genotypes at cold conditions (Fig. 6a). In contrast, a gene encoding a *pyrophosphate-energized vacuolar membrane proton pump 1* (Zm00001d037492) displayed higher expression levels in the genotypes with faster root growth at cold conditions (Fig. 6b) and was in general highly expressed taking into account expression across all genotypes (average read count per sample: 12,424).

In the set of 563 genes associated with growth rate at cold conditions, we detected 29 enriched GO terms (Table S9). Similar to the GO networks of the genes associated with cold tolerance, two connected networks were associated with amino acid transport and catabolic processes (Fig. 6c).

## Discussion

In this study, we investigated the genetic diversity for cold tolerance of the European maize flint landrace “Petkuser Ferdinand rot” during early root development. The rationale to survey cold tolerance was that improvement of this trait will allow earlier planting for increased biomass production. At the same time, as a consequence of earlier planting the risk of potential summer drought will be mitigated by an earlier onset of flowering.

Maize landraces are a rich source of favorable alleles for broadening the genetic base of elite germplasm [4]. We hypothesized that European maize landraces display substantial variation for cold tolerance thus carrying beneficial alleles for this trait which might not be present in elite material. This notion is supported by a study which demonstrated that in a panel of 35 European maize flint landraces including “Petkuser Ferdinand rot” used in this study, one landrace covers at least 75% of the genomic variation of all landraces present in this panel [27]. Phenotypic variation in the DH population derived from the European maize flint landrace “Petkuser Ferdinand rot” used in this study was demonstrated by > 4-fold changes in cold tolerance of root growth (Fig. 1). Similarly, extensive diversity for cold tolerance during early development has been observed in other maize landraces for aboveground traits [7, 28–30].

### Transcriptomic diversity with respect to cold tolerance

An RNA-seq experiment resulted in 19,917 to 21,011 active genes in the 21 DH genotypes derived from the European landrace “Petkuser Ferdinand rot” (Fig. S1). In a study surveying a diverse set of seven US inbred lines at a similar stage of root development between 24,923–25,149 genes were active [31]. The lower mapping rate in this study is likely the consequence of mapping the transcriptomes of these Northern flint genotypes on the high quality reference genome sequence of the dent inbred line B73 [32].

In the principal component analysis (Fig. 2), samples from the same genotype subjected to cold and control treatment clustered closely together while cold tolerant and cold susceptible genotypes were not separated clearly, except for the two most cold tolerant lines (PE0002 and PE0161). This indicates that the transcriptomic variation observed in our study mainly reflects general genotypic differences and only to a minor degree differences in cold tolerance or cold treatment effects. Substantial genetic variability between genotypes in contrast to treatment effects, as observed in our experiments, was also described in previous studies on the diversity of European flint landraces accessed through doubled haploids [4, 8, 27]. Similar results were obtained in a set of diverse European flint and dent inbred lines subjected to heat stress where the transcriptomic responses between heat tolerant and susceptible genotypes were highly divergent [33]. Likewise, in a transcriptome study with the US inbred lines B73 and Mo17 and their reciprocal F_1_–hybrids genotypic differences were much more pronounced than differences between water deficit stress and control conditions (Marcon et al., 2017).

The substantial transcriptomic variation between genotypes in this study provided the opportunity to evaluate the response to cold stress in a genetically diverse germplasm. In contrast, previous transcriptome studies on abiotic stress tolerance such as waterlogging, cold stress, drought stress typically focused on only one or a few maize inbred lines or hybrids under different conditions [15, 34–40].

### Transcriptomic response upon cold treatment

We investigated the effect of mild cold stress on the transcriptomes of 21 DH-lines. Cold responsive genes were defined as genes differentially expressed between control (18 °C) and cold (12 °C) night temperatures. We identified 22 cold responsive genes which were differentially regulated in ≥15 of the 21 surveyed genotypes (Fig. 4a). Among these, the *heat stress transcription factor C-1* (Zm00001d039166) was significantly upregulated upon cold treatment in 20 of 21 genotypes (Fig. 4b). In previous studies this gene was differentially expressed at severe cold treatment and heat stress [41] as well as at a variety of water deficit conditions [35, 42]. Hence, this transcription factor responds to several abiotic stress types and might regulate a plethora of downstream target genes. Similarly, the *cysteine oxidase 1* (Zm00001d039166), which was downregulated at cold treatment in 19 of 21 genotypes in this study, is also regulated by several other abiotic stress factors such as submergence [37] water deficit [42] and heat stress [41].

GO term enrichment of the 148 genes differentially expressed upon cold treatment, allowed for the identification of molecular processes associated with this type of abiotic stress (Table S4). In total, 20 of 59 enriched GO terms were associated with responses to different abiotic stresses including the responses to light, heat and phytohormones (Table S4). Different abiotic stresses often result in similar responses in plants. They are frequently based on oxidative stress associated with reactive oxygen species such as H_2_O_2_, which serve as inducers of tolerances to abiotic and biotic stresses [43]. GO terms associated with responses to several abiotic stresses were also enriched in an experiment with diverse European maize germplasm response to heat stress [33].

The most enriched GO terms in our dataset associated with response to abiotic stresses were “response to hypoxia”, “reponse to karrikin” and “response to cold” (Table S4). Using REVIGO we were able to identify networks of enriched GO terms with high similarity between each other (Fig. 4d). This connected network grouped around the term “response to cold” as the term with highest relevance. The GO terms associated with abiotic responses, which were connected with the “response to cold” were the responses to water deprivation (i.e. drought) and to karrikin. In previous studies, crosstalk between the signaling pathways of drought and cold stress were suggested [44] because many genes are induced by both abiotic stresses. Similarly, an association of cold response with the response to karrikin was observed in *Camellia sinensis* [45]. Plant-derived smoke, which is the main source of natural karrikin, increases seed germination and the length and fresh weight of maize seedlings, but also regulates reactive oxygen species and their scavenging system [46].

### Transcriptomic responses associated with cold tolerance

To investigate the relative primary root growth of the surveyed maize genotypes under control versus cold conditions we determined cold tolerance as the ratio of root growth at cold versus control conditions (Fig. 1b). In total, 3254 genes were associated with this type of cold tolerance. Among those, several genes encoded transcription factors such as *bHLH-transcription factor 136* (Zm00001d021019; Fig. 5a), *GRAS-transcription factor 82* (Zm00001d048682) (Fig. 5b) and *Aux/IAA transcription factor 14* (Zm00001d049141; Fig. 5c). This observation is in line with the notion that a multitude of transcription factors is involved in the regulation of gene expression upon abiotic stress such as cold [47]. Genotype specific expression of *Aux/IAA transcription factor 14* (Zm00001d049141) upon cold and heat stress has also been observed between the genotypes B73 and Mo17 [41]. GRAS-transcription factors like Zm00001d048682 (Fig. 5b) have been demonstrated to be involved in the regulation of root and shoot development and in the improvement of plant resistance to abiotic stresses [48]. Genotype-specific upregulation of this gene upon cold stress has been demonstrated for several genotypes [41], although in the present study we did not observe differential regulation upon cold stress, but rather higher expression in cold tolerant genotypes (Fig. 5b).

The subset of 10 cold tolerance associated genes which were uniquely expressed in the one, two or three most tolerant DH-lines (Fig. 5d), might be of special interest for functional characterization, as they might be associated with increased cold tolerance. Remarkably, six out of ten genes have no associated function yet. Our results suggest that these ten genes are highly variable between genotypes. For the gene Zm00001d031037 (Fig. 5d), this is supported by expression analyses of maize leaves of eight diverse inbred lines, where it was expressed in four lines but not in the other four lines [49]. In the single DH-line, where Zm00001d031037 was expressed in our experiment, it was significantly downregulated at cold conditions. Concordantly, Zm00001d031037 was shown to be upregulated at heat in inbred line Mo17 and downregulated at cold in Mo17 and Oh43, but not differentially expressed at either condition in B73 [41].

From the GO terms enriched in the set of genes associated with cold tolerance (Fig. 5e), we identified three networks of connected GO terms with high similarity among each other. The top enriched GO term was “cellular oxidant detoxification” (GO:0098869) (Table S7) which is associated with the reduction of the toxicity of superoxide radicals or hydrogen peroxide. These molecules are associated with oxidative stress occurring when plants are subjected to stresses such as drought, heat or cold.

### Transcriptomic patterns associated with growth rate at cold

Another measure of cold performance is the growth rate in cm/day of seedling roots at cold conditions (Fig. 1c). This parameter is of agricultural relevance because it determines the performance of a genotype during early stages of growth under cold spring conditions.

Among the genes related to the trait growth rate at cold, the *EXORDIUM* protein coding gene (Zm00001d018106) showed consistently low expression in genotypes with high growth rates at cold conditions (Fig. 6a). In previous studies it was demonstrated that this gene is strongly induced by multiple biotic and abiotic stress types including cold and heat [41], submergence [37] and fungal infections [50]. EXORDIUM putatively promotes shoot and root growth in plants [51]. This protein might therefore play a role in the maintenance of growth under adverse environmental conditions in stress tolerant plants to avoid fatal tissue damage.

Among the GO terms enriched in the set of genes associated with growth rate at cold (Fig. 6c) but also with cold tolerance (Fig. 5e) was amino acid transport. Some amino acids can act as precursors for antioxidants or osmolytes or act themselves as osmolytes to prevent the deleterious effect of cold stress. Antioxidants such as glutathione are involved in the response pathways to cold and also in the tolerance to cold as observed in *Pinus halapensis* seedlings [52]. Glutathione was enriched in cold tolerant seedlings in comparison with cold sensitive seedlings [52]. Furthermore amino acids could act themselves as osmolytes to prevent damages from cold stress [52].

### Association between cold tolerance and growth rate at cold

The order of the genotypes with respect to growth rate at cold conditions (Fig. 1c) was different from the order with respect to cold tolerance (Fig. 1b). However, the two parameters of the 276 lines from the ‘Petkuser Ferdinand rot’ DH population were correlated significantly (*p* < 0.001) with a low coefficient of correlation of *r*  = 0.22. This implies that genotypes which perform better at cold conditions are also in general more cold tolerant. However, the low coefficient of correlation between the two traits in the DH-population also suggests that the genetic control of these traits is partly independent from each other. This allows combination of both traits during breeding.

## Conclusion

The European flint germplasm surveyed in this study showed high variation in their phenotypic and transcriptomic behavior associated with response to cold treatment, cold tolerance and growth rate at cold. Genetic analyses of mutants defective in the cold related genes identified in this study will help to better understand the molecular principles underlying this important trait during early seedling development. This might pave the way for introgressing beneficial alleles of these genes into modern maize by recurrent selection or genome editing thus improving cold tolerance.

## Methods

### Selection of DH lines by phenotypic assessment

We selected 21 doubled haploid (DH) lines from 276 genotypes of a DH population of the flint landrace ‘Petkuser Ferdinand rot’ [27, 53] based on seed availability. This population was generated by the in vivo haploid induction method [54]. The landrace ‘Petkuser Ferdinand rot’, originating from Northeastern Germany, has been obtained from the federal ex situ Gene Bank for Agricultural and Horticultural Crop Species at the Leibniz Institute of Plant Genetics and Crop Plant Research, Gatersleben, Germany.

Maize roots were imaged after growing five seedlings per DH line under control conditions (26 °C/18 °C for 16 h light/8 h dark) for 4 days and subsequently for 2 days either under control or under cold (16 °C /12 °C for 16 h light/8 h dark) treatment to simulate diverging soil temperatures during early establishment of the seedlings. Seedlings were grown in germination paper rolls (Anchor Paper Co, Saint Paul, USA) as previously described [31, 55]. Germination paper rolls were randomly arranged in 10 L buckets filled with ca. 3 L distilled water.

We used GiA Roots [56] to determine root network perimeter and calculated root growth-rates by computing a linear regression of root network perimeter from the images before and after treatment. We defined the cold tolerance of each genotype as

Cold tolerance = growth rate at cold/growth rate at control.

We selected eleven cold tolerant DH-lines and ten cold susceptible DH-lines based on cold tolerance values, germination rate and availability of seeds for our transcriptome experiment.

### Plant growth and RNA isolation

For each biological replicate, 10 seedlings were grown as described above, for 4 days under control condition (26 °C/18 °C for 16 h light/8 h dark). In this second experiment, the pre-selected DH-lines were only subjected to a treatment (12 °C cold and 18 °C control) period of 8 h, instead of 2 days. The mild cold treatment of 12 °C was chosen to prevent strong damage to root tissue observed below 10 °C [9], but to still induce physiological reactions typically occurring at cold stress. After treatment, root tips of ten primary roots comprising the meristematic and elongation zone (tip of the root excluding root hair zone, with maximal length of 1 cm) were excised with a razor blade, pooled, immediately frozen in liquid nitrogen and stored at − 80 °C until RNA extraction.

We prepared 168 RNA samples representing 21 genotypes, two treatments (cold and control) and four biological replicates per genotype by treatment combination. Total RNA was isolated using the RNeasy Plant Mini Kit (Qiagen, Hilden, Germany) including on-column DNA removal. RNA quantity and quality were assessed using the 2100 Bioanalyzer and Agilent RNA 6000 Nano Chip (Agilent Technologies, Böblingen, Germany). For all samples, RIN (RNA integrity number, [57]) values ≥8 indicated their high quality and integrity.

### RNA-sequencing

cDNA libraries for RNA-seq were constructed according to the TruSeq RNA sample preparation protocol (Illumina, San Diego CA, USA). Library indexing, cluster preparation, and paired-end sequencing were performed according to the manufacturer’s instructions (Illumina). Sequencing of 100 bp paired-end reads was performed on an Illumina HiSeq 4000 platform.

### RNA-seq data processing

RNA-seq raw reads were quality trimmed using Trimmomatic (version 0.36, [58]) according to the manual with the parameters for paired-end reads ‘ILLUMINACLIP:TruSeq3-PE-2.fa:3:30:10 LEADING:3 TRAILING:3 MAXINFO:30:0.8 MINLEN:40’.

The trimmed reads were aligned to the B73 reference genome sequence AGPv.4 (https://www.maizegdb.org/assembly) using Hisat2 (version 2.1.0, [59]) according to the manual with the parameters for paired-end reads ‘--min-intronlen 20 --max-intronlen 60000 --known-splicesites-infile prepared-splice-sites-file’. The file containing the splice sites information was prepared using the Python script hisat2_extract_splice_sites.py included in the Hisat2 binary distribution and an index of the reference sequence assembly was created using hisat2-build.

The software samtools (version 1.3.1, [60]) was applied to transform file format .sam to .bam, to order the aligned reads according to their position and divide paired and unpaired read alignments. Reads were counted when they aligned unambiguously with an alignment quality score of > 10 to an exon of a gene as specified in the AGPv4 annotation file (Ensembl release 36, 06/27/17, ftp://ftp.ensemblgenomes.org/pub/plants/release-36/gff3/zea_mays). Alignment of sequences to the reference genome was performed using HTSeq (version 0.10.0, [61]) with the parameters ‘-r pos -t exon -i gene_id -s no --secondary-alignments ignore --supplementary-alignments ignore’.

Count tables were produced separately for paired and unpaired read alignments. Count tables were combined by sample adding counts per feature (gene) creating a single count table with 168 samples and 46,272 genes. We removed genes with a sum of less than ten reads across all samples from further analyses.

### Principal component analysis

A principal component analysis (PCA) was performed on the expression data using the normalization procedure rlog() implemented in the R package DESeq2 (version 1.18.1, [62]). The plotPCA() function was used for plotting of the data.

### Expression analysis

We normalized expression values with library size by calculating fragments per million (FPM) reads using the fpm() function of DESeq2. We declared genes with a mean FPM across samples of a genotype ≥1 as expressed.

### Differential gene expression

Expression levels of genes were estimated by the variance-mean dependence in the count table based on a generalized linear model using the negative binomial distribution within the R package DESeq2 [62] calculating log_2_ fold change (log_2_FC) values between groups of samples specified in the following paragraph. Significance values for log_2_FC values were calculated as Wald test *p*-value and were adjusted by the Benjamini-Hochberg procedure to obtain false discovery rates (FDR) [63]. To illustrate expression levels of selected genes, raw counts were normalized with DESeq’s built-in function plotCounts().

We applied several models to identify differentially expressed genes. First, we used a model with three factors including data of all samples,

1 $$ Y_{ij} = \mu + c_{i} + t{j} + {(ct)}_{ij} + {\varepsilon}_{ij}, $$

where *Y*_*ij*_ was the expression of respective gene of DH-line *i* at treatment level *j*. *μ* was the general mean, *c*_*i*_ was defined as the cold tolerance of DH-line *i* as define above*. t*_*j*_ was the treatment level *j* (cold and control) and *(ct)*_*ij*_ was the interaction of cold tolerance and the treatment level. *ε*_*ij*_ was the residual error term.

The second model was used with samples of each genotype separately to identify genes which are differentially expressed between cold and control conditions in the respective genotype.

2 $$ {Y}_j=\upmu +{t}_j+{\upvarepsilon}_j, $$

where *Y*_*j*_ was the expression of respective gene at treatment level *j*, *μ* was the general mean, *t*_*j*_ the treatment level *j* (cold and control) and *ε*_*j*_ was the residual error term.

In the third model, we input all samples at cold conditions excluding samples at control conditions.

3 $$ {Y}_i=\upmu +{g}_i+{\upvarepsilon}_i, $$

where *Y*_*i*_ was the expression of respective gene of DH-line *i*, *μ* was the general mean, *g*_*i*_ the growth rate of DH-line *i* at cold conditions and *ε*_*j*_ was the residual error term. With this model, we explained gene expression with relative growth rate of a genotype at cold conditions.

### Gene ontology term enrichment

We tested significant (*p* < 0.05) enrichment of gene ontology (GO) terms in the gene sets assembled using the differential gene expression analysis with FDR < 5% and log_2_FC of > 1 or < − 1, with model (1) with factor treatment and factor cold tolerance as well as with model (3) with factor growth rate at cold [64, 65]. GO-terms were accessed from the GAMER annotation set for the reference genome sequence AGPv4, a set with high gene coverage for the maize inbred line B73 [66]. We filtered the GAMER annotation for genes expressed in at least one genotype with at least one fragment per million reads on average across all 168 samples (24,448 out of 46,272 annotated genes). The R-package GO.db [67] was used to obtain the biological descriptions for each GO term. The R-package topGO [68] was used to perform weighted Fisher’s exact test on the GO terms present in the respective gene set. We pruned the GO hierarchy from terms with less than five annotated genes and used only GO terms associated to biological processes. We did not consider expression values, log_2_FC or FDR values from the differential expression analysis to ensure consistency of the method throughout the study [68]. For each gene set, significantly (*p* < 0.01) enriched GO terms were then evaluated using REVIGO (http://revigo.irb.hr/; [69]). Functional redundancies were reduced and the results were visualized based on the semantic similarity, relying on the ‘most informative common ancestor’ approach. The allowed similarity was set to 0.7 (medium). *Zea mays* was selected as database, GO term size and all other parameters were left at their default values. Using REVIGO we were able to assess associations and connections between different GO terms enriched in the gene sets and visualize them with network graphs. We visualized the networks using Cytoscape [70], where *p*-values derived from topGO’s enrichment procedure, frequency of the respective term in the database and similarities of the enriched GO terms were depicted as colors and size of connected bubbles (nodes). The placement of the nodes was adjusted to fit the respective figures and does not provide additional information.

## Supplementary information

**Additional file 1: Figure S1:** Number of genes active (expressed on average at ≥1 fragment per million reads (FPM)) in at least one genotype (black, 24,448 genes) and in individual genotypes (colors). Core transcripts (17,204 genes) expressed in all genotypes, are depicted with transparent coloring. Color code according to Fig. 1.

**Additional file 2: Table S1:** Overview of the sample distribution within genotype, treatment, biological replication, RNA-Seq output, and mapping results.

**Additional file 3: Table S2:** Characteristics of the 148 genes differentially expressed between cold and control conditions (see Fig. 3).

**Additional file 4: Table S3:** Up- (1) or down regulated (− 1) genes differentially expressed between cold and control conditions in the 21 genotypes. No differential expression is depicted as 0. Sum indicates the sum of differential expression scores (1, 0, − 1) across genotypes, sum tolerant lines and sum susceptible lines are the sums of all genotypes in the respective class.

**Additional file 5: Table S4:** Gene ontology (GO) terms enriched (*p* < 0.05) in the 148 genes differentially expressed between cold and control conditions (see Table S2).

**Additional file 6: Table S5:** Characteristics of the 3254 cold tolerant genes (see Fig. 3).

**Additional file 7: Table S6:** Characteristics of genes exclusively expressed in the three most cold tolerant genotypes (see Fig. 5d).

**Additional file 8: Table S7:** Gene ontology (GO) terms enriched (*p* < 0.05) in the 3254 genes associated with cold tolerance (see Table S5).

**Additional file 9: Table S8:** Characteristics of the 563 genes associated to growth rate at cold conditions (cf. Fig. 4).

**Additional file 10: Table S9:** Gene ontology (GO) terms enriched (*p* < 0.05) in genes associated with growth rate at cold conditions (see Table 9).

## Data Availability

The raw sequencing data has been deposited in the NCBI sequencing read archive (SRA; http://www.ncbi.nlm.nih.gov/sra; BioProject accession number PRJNA556806). The datasets supporting the conclusions of this article are included within the article and its additional files.

## Abbreviations

DH: Doubled-haploid
FC: Fold change
GO: Gene ontology
FDR: False discovery rate
PCA: Principal component analysis

## References

[1] Hake S, Ross-Ibarra J (2015). Genetic, evolutionary and plant breeding insights from the domestication of maize. eLife.

[2] FAO. FAOSTAT: crops. http://www.fao.org/faostat/en/#data/QC. 2018.

[3] Eurostat. *Green maize by area, production and humidity*. https://ec.europa.eu/eurostat/web/products-datasets/-/tag00101. 2018.

[4] Sood S, Flint-Garcia S, Willcox MC, Holland JB, Tuberosa R, Graner A, Frison E (2014). Mining natural variation for maize improvement: selection on phenotypes and genes. Genomics of Plant Genetic Resources: Volume 1. Managing, Sequencing and Mining Genetic Resources.

[5] Tenaillon MI, Charcosset A (2011). A European perspective on maize history. C R Biol.

[6] Haberer G, Bauer E, Kamal N, Gundlach H, Fischer I, Seidel MA, Spannagl M, Marcon C, Ruban A, Urbany C, Nemri A, Hochholdinger F, Ouzunova M, Houben A, Schön C-C, Mayer KFX. European maize genomes unveil pan-genomic dynamics of repeats and genes. bioRxiv. 2019;766444.

[7] Rodríguez VM, Romay MC, Ordás A, Revilla P (2010). Evaluation of European maize (*Zea mays* L.) germplasm under cold conditions. Genet Resour Crop Evol.

[8] Strigens A, Schipprack W, Reif JC, Melchinger AE (2013). Unlocking the genetic diversity of maize landraces with doubled haploids opens new avenues for breeding. PLoS One.

[9] Greaves JA (1996). Improving suboptimal temperature tolerance in maize - the search for variation. J Exp Bot.

[10] Haldimann P (1998). Low growth temperature-induced changes to pigment composition and photosynthesis in *Zea mays* genotypes differing in chilling sensitivity. Plant Cell Environ.

[11] Foyer CH, Vanacker H, Gomez LD, Harbinson J (2002). Regulation of photosynthesis and antioxidant metabolism in maize leaves at optimal and chilling temperatures. Plant Physiol Biochem.

[12] Rymen B, Fiorani F, Kartal F, Vandepoele K, Inze D, Beemster GTS (2007). Cold nights impair leaf growth and cell cycle progression in maize through transcriptional changes of cell cycle genes. Plant Physiol.

[13] Sobkowiak A, Jończyk M, Jarochowska E, Biecek P, Trzcinska-Danielewicz J, Leipner J, Fronk J, Sowiński P (2014). Genome-wide transcriptomic analysis of response to low temperature reveals candidate genes determining divergent cold-sensitivity of maize inbred lines. Plant Mol Biol.

[14] Farooq M, Aziz T, Wahid A, Lee D-J, Siddique KHM (2009). Chilling tolerance in maize: agronomic and physiological approaches. Crop Pasture Sci.

[15] Mao J, Yu Y, Yang J, Li G, Li C, Qi X, Wen T, Hu J (2017). Comparative transcriptome analysis of sweet corn seedlings under low-temperature stress. Crop J.

[16] Battal P, Erez ME, Turker M, Berber I (2008). Molecular and physiological changes in maize (*Zea mays*) induced by exogenous NAA, ABA and MeJa during cold stress. Ann Bot Fenn.

[17] Khan MIR, Fatma M, Per TS, Anjum NA, Khan NA (2015). Salicylic acid-induced abiotic stress tolerance and underlying mechanisms in plants. Front Plant Sci.

[18] Aroca R, Tognoni F, Irigoyen JJ, Sánchez-Díaz M, Pardossi A (2001). Different root low temperature response of two maize genotypes differing in chilling sensitivity. Plant Physiol Biochem.

[19] Melkonian J, Yu LX, Setter TL (2004). Chilling responses of maize (*Zea mays* L.) seedlings: root hydraulic conductance, abscisic acid, and stomatal conductance. J Exp Bot.

[20] Nagel KA, Kastenholz B, Jahnke S, van Dusschoten D, Aach T, Mühlich M, Truhn D, Scharr H, Terjung S, Walter A, Schurr U (2009). Temperature responses of roots: impact on growth, root system architecture and implications for phenotyping. Funct Plant Biol.

[21] Breitkopf A. Monatliche Durchschnittstemperatur in Deutschland von Juni 2018 bis Juni 2019. https://de.statista.com/statistik/daten/studie/5564/umfrage/monatliche-durchschnittstemperatur-in-deutschland/Statista. 2019.

[22] Landwirtschaftskammer Nordrhein-Westfalen. Saatzeit für Mais. https://www.landwirtschaftskammer.de/landwirtschaft/ackerbau/mais/saatzeit-pdf.pdf. 2015.

[23] Mock JJ, McNeill MJ (1979). Cold tolerance of maize inbred lines adapted to various latitudes in North America. Crop Sci.

[24] Hund A, Fracheboud Y, Soldati A, Frascaroli E, Salvi S, Stamp P (2004). QTL controlling root and shoot traits of maize seedlings under cold stress. Theor Appl Genet.

[25] Arshad MA, Azooz RH (1996). Tillage effects on soil thermal properties in a semiarid cold region. Soil Sci Soc Am J.

[26] Chaikam V, Molenaar W, Melchinger AE, Boddupalli PM (2019). Doubled haploid technology for line development in maize: technical advances and prospects. Theor Appl Genet.

[27] Hölker AC, Mayer M, Presterl T, Bolduan T, Bauer E, Ordas B, Brauner PC, Ouzunova M, Melchinger AE, Schön CC (2019). European maize landraces made accessible for plant breeding and genome-based studies. Theor Appl Genet.

[28] Revilla P, Boyat A, Álvarez A, Gouesnard B, Ordás B, Rodríguez VM, Ordás A, Malvar RA (2006). 2006. Contribution of autochthonous maize populations for adaptation to European conditions. Euphytica.

[29] Peter R, Eschholz TW, Stamp P, Liedgens M (2009). Swiss Flint maize landraces - a rich pool of variability for early vigour in cool environments. Field Crops Res.

[30] Schneider DN, Freitag NM, Liedgens M, Feil B, Stamp P (2011). Early growth of field-grown swiss flint maize landraces. Maydica.

[31] Baldauf JA, Marcon C, Lithio A, Vedder L, Altrogge L, Piepho HP, Schoof H, Nettleton D, Hochholdinger F (2018). Single-parent expression is a general mechanism driving extensive complementation of non-syntenic genes in maize hybrids. Curr Biol.

[32] Jiao Y, Peluso P, Shi J, Liang T, Stitzer MC, Wang B, Campbell MS, Stein JC, Wei X, Chin CS, Guill K, Regulski M, Kumari S, Olson A, Gent J, Schneider KL, Wolfgruber TK, May MR, Springer NM, Antoniou E, McCombie WR, Presting GG, McMullen M, Ross-Ibarra J, Dawe RK, Hastie A, Rank DR, Ware D (2017). Improved maize reference genome with single-molecule technologies. Nature.

[33] Frey FP, Urbany C, Hüttel B, Reinhardt R, Stich B (2015). Genome-wide expression profiling and phenotypic evaluation of European maize inbreds at seedling stage in response to heat stress. BMC Genomics.

[34] Kollipara KP, Saab IN, Wych RD, Lauer MJ, Singletary GW (2002). Expression profiling of reciprocal maize hybrids divergent for cold germination and desiccation tolerance. Plant Physiol.

[35] Opitz N, Marcon C, Paschold A, Malik WA, Lithio A, Brandt R, Piepho HP, Nettleton D, Hochholdinger F (2016). Extensive tissue-specific transcriptomic plasticity in maize primary roots upon water deficit. J Exp Bot.

[36] Opitz N, Paschold A, Marcon C, Malik WA, Lanz C, Piepho HP, Hochholdinger F (2014). Transcriptomic complexity in young maize primary roots in response to low water potentials. BMC Genomics.

[37] Campbell MT, Proctor CA, Dou Y, Schmitz AJ, Phansak P, Kruger GR, Zhang C, Walia H (2015). Genetic and molecular characterization of submergence response identifies subtol6 as a major submergence tolerance locus in maize. PLoS One.

[38] Wang B, Tseng E, Regulski M, Clark TA, Hon T, Jiao Y, Lu Z, Olson A, Stein JC, Ware D (2016). Unveiling the complexity of the maize transcriptome by single-molecule long-read sequencing. Nat Commun.

[39] Arora K, Panda KK, Mittal S, Mallikarjuna MG (2017). RNAseq revealed the important gene pathways controlling adaptive mechanisms under waterlogged stress in maize. Sci Rep.

[40] Di Fenza M, Hogg B, Grant J, Barth S (2017). Transcriptomic response of maize primary roots to low temperatures at seedling emergence. PeerJ.

[41] Makarevitch I, Waters AJ, West PT, Stitzer M, Hirsch CN, Ross-Ibarra J, Springer NM (2015). Transposable elements contribute to activation of maize genes in response to abiotic stress. PLoS Genet.

[42] Tai H, Lu X, Opitz N, Marcon C, Paschold A, Lithio A, Nettleton D, Hochholdinger F (2016). Transcriptomic and anatomical complexity of primary, seminal, and crown roots highlight root type-specific functional diversity in maize (*Zea mays* L.). J Exp Bot.

[43] Desikan R (2001). A.-H.-Mackerness S, Hancock JT, Neill SJ. Regulation of the Arabidopsis transcriptome by oxidative stress. Plant Physiol.

[44] Shinozaki K, Yamaguchi-Shinozaki K, Seki M (2003). Regulatory network of gene expression in the drought and cold stress responses. Current Opin Plant Biol.

[45] Li N, Yue C, Cao H, Qian W, Hao X, Wang Y, Wang L, Ding C, Wang X, Yang Y (2018). Transcriptome sequencing dissection of the mechanisms underlying differential cold sensitivity in young and mature leaves of the tea plant (*Camellia sinensis*). J Plant Physiol.

[46] Aslam MM, Rehman S, Khatoon A, Jamil M, Yamaguchi H, Hitachi K, Tsuchida K, Li X, Sunohara Y, Matsumoto H, Komatsu S (2019). Molecular responses of maize shoot to a plant derived smoke solution. Int J Mol Sci.

[47] Kimotho RN, Baillo EH, Zhang Z (2019). Transcription factors involved in abiotic stress responses in Maize (*Zea mays* L.) and their roles in enhanced productivity in the post genomics era. PeerJ.

[48] Zhang B, Liu J, Yang ZE, Chen EY, Zhang CJ, Zhang XY, Li FG (2018). Genome-wide analysis of GRAS transcription factor gene family in *Gossypium hirsutum* L. BMC Genomics.

[49] Baute J, Herman D, Coppens F, De Block J, Slabbinck B, Dell’Acqua M, Pè ME, Maere S, Nelissen H, Inzé D (2015). Correlation analysis of the transcriptome of growing leaves with mature leaf parameters in a maize RIL population. Genome Biol.

[50] Lanubile A, Ferrarini A, Maschietto V, Delledonne M, Marocco A, Bellin D (2014). Functional genomic analysis of constitutive and inducible defense responses to *Fusarium verticillioides* infection in maize genotypes with contrasting ear rot resistance. BMC Genomics.

[51] Coll-Garcia D, Mazuch J, Altmann T, Müssig C (2004). *EXORDIUM* regulates brassinosteroid-responsive genes. FEBS Lett.

[52] Taïbi K, Del Campo AD, Vilagrosa A, Bellés JM, López-Gresa MP, López-Nicolás JM, Mulet JM (2018). Distinctive physiological and molecular responses to cold stress among cold-tolerant and cold-sensitive *Pinus halepensis* seed sources. BMC Plant Biol.

[53] Mayer M, Unterseer S, Bauer E, de Leon N, Ordas B, Schön C-C (2017). Is there an optimum level of diversity in utilization of genetic resources?. Theor Appl Genet.

[54] Röber FK, Gordillo GA, Geiger HH (2005). *In vivo* haploid induction in maize - performance of new inducers and significance of doubled haploid lines in hybrid breeding. Maydica.

[55] Hoecker N, Keller B, Piepho HP, Hochholdinger F (2006). Manifestation of heterosis during early maize (*Zea mays* L.) root development. Theor Appl Genet.

[56] Galkovskyi T, Mileyko Y, Bucksch A, Moore B, Symonova O, Price CA, Topp CN, Iyer-pascuzzi AS, Zurek PR, Fang S, Harer J, Benfey PN, Weitz JS (2012). GiA roots: software for the high throughput analysis of plant root system architecture. BMC Plant Biol.

[57] Schroeder A, Mueller O, Stocker S, Salowsky R, Leiber M, Gassmann M, Lightfoot S, Menzel W, Granzow M, Ragg T. The RIN: An RNA integrity number for assigning integrity values to RNA measurements. BMC Mol Biol 2006;7, 1–14. 10.1186/1471-2199-7-3 PMC1413964 16448564

[58] Bolger AM, Lohse M, Usadel B (2014). Trimmomatic: a flexible trimmer for Illumina sequence data. Bioinformatics.

[59] Kim D, Langmead B, Salzberg SL (2015). HISAT: a fast spliced aligner with low memory requirements. Nat Methods.

[60] Li H, Handsaker B, Wysoker A, Fennell T, Ruan J, Homer N, Marth G, Abecasis G, Durbin R (2009). The sequence alignment/map format and SAMtools. Bioinformatics.

[61] Anders S, Pyl PT, Huber W (2015). HTSeq-A Python framework to work with high-throughput sequencing data. Bioinformatics.

[62] Love MI, Huber W, Anders S (2014). Moderated estimation of fold change and dispersion for RNA-seq data with DESeq2. Genome Biol.

[63] Benjamini Y, Hochberg Y (1995). Controlling the false discovery rate: a practical and powerful approach to multiple testing. J R Stat Soc Series B Stat Methodol.

[64] Ashburner M, Ball CA, Blake JA, Botstein D, Butler H, Cherry JM, Davis AP, Dolinski K, Dwight SS, Eppig JT, Harris MA (2000). Gene ontology: tool for the unification of biology. Nat Genet.

[65] The Gene Ontology Consortium (2019). The gene ontology resource: 20 years and still GOing strong. Nucleic Acids Res.

[66] Wimalanathan K, Friedberg I, Andorf CM, Lawrence-Dill CJ (2018). Maize GO annotation - methods, evaluation, and review (maize-GAMER). Plant Direct.

[67] Carlson M, Falcon S, Pages H, Li N. GO. db: A set of annotation maps describing the entire Gene Ontology. R package version, 3.7.0. 2018.

[68] Alexa AA, Rahnenfuhrer J, Alexa MA. Package ‘topGO’ - Enrichment analysis for gene ontology. R package version 2.37.0. 2019.

[69] Supek F, Bošnjak M, Škunca N, Tomislav Š (2011). REVIGO summarizes and visualizes long lists of gene ontology terms. PLoS One.

[70] Shannon P, Markiel A, Ozier O, Baliga NS, Wang JT, Ramage D, Amin N, Schwikowski B, Ideker T (2003). Cytoscape: a software environment for integrated models of biomolecular interaction networks. Genome Res.

