# Supplementary figures and images for: Transcriptomic diversity in seedling roots of European flint maize in response to cold
Source: BMC Genomics. 2020 Apr 15;21:300. doi: 10.1186/s12864-020-6682-1 (PMC7158136; doi:10.1186/s12864-020-6682-1)

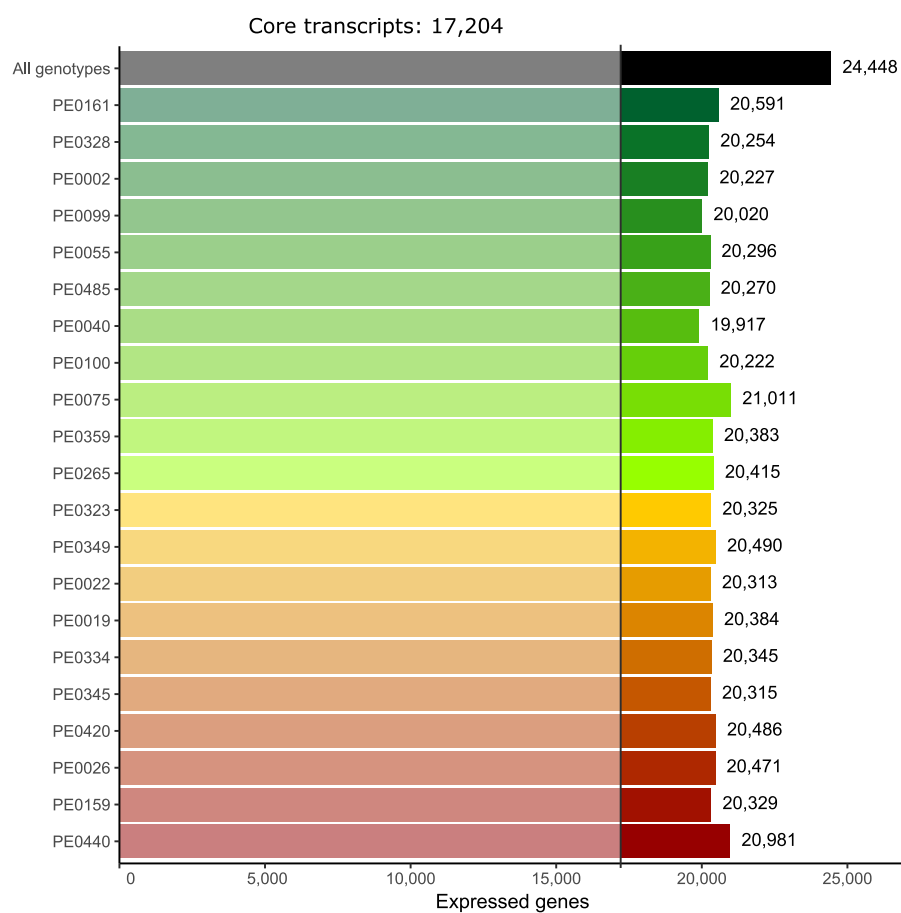

Figure S1

Supplement: Supplementary file 1 — Additional file 1: Figure S1: Number of genes active (expressed on average at ≥1 fragment per million reads (FPM)) in at least one genotype (black, 24,448 genes) and in individual genotypes (colors). Core transcripts (17,204 genes) expressed in all genotypes, are depicted with transparent coloring. Color code according to Fig. 1. [file 12864_2020_6682_MOESM1_ESM.pdf]
